# A Phenomenological Approach to Medication Adherence in Elderly Patients: A Qualitative Study

**DOI:** 10.3390/healthcare12191925

**Published:** 2024-09-25

**Authors:** Gloria Liquori, Nicolò Panattoni, Aurora De Leo, Sara Dionisi, Noemi Giannetta, Beatrice Gasperi, Giovanni Battista Orsi, Flavio Di Muzio, Marco Di Muzio, Emanuele Di Simone

**Affiliations:** 1 Department of Biomedicine and Prevention, University of Rome Tor Vergata, 00128 Rome, Italy; gloria.liquori@uniroma1.it (G.L.); beatrice.gasperi@students.uniroma2.eu (B.G.); 2 Department of Public Health and Infectious Diseases, Sapienza University of Rome, 00185 Rome, Italy; nicolo.panattoni@uniroma1.it (N.P.); giovanni.orsi@uniroma1.it (G.B.O.); emanuele.disimone@uniroma1.it (E.D.S.); 3 Nursing Research Unit IFO, IRCCS Istituti Fisioterapici Ospitalieri, 00144 Rome, Italy; aurora.deleo1975@gmail.com; 4 Nursing, Technical, Rehabilitation Department, DaTeR Azienda Unità Sanitaria Locale di Bologna, 40121 Bologna, Italy; sara.dionisi@uniroma1.it; 5 Departmental Faculty of Medicine, Saint Camillus International University of Health and Medical Sciences (UniCamillus), 00131 Rome, Italy; noemi.giannetta@unicamillus.org; 6 Azienda Sanitaria Locale Roma 4, 00053 Rome, Italy; flaviodimuzio@yahoo.it; 7 Department of Clinical and Molecular Medicine, Sapienza University of Rome, 00185 Rome, Italy

**Keywords:** medication adherence, polypharmacy, elderly patients, phenomenological approach, qualitative research

## Abstract

**Background:** Patient adherence to drug treatment is crucial to the success of any prescribed therapy, especially in chronic conditions. The present phenomenological qualitative study aims to explore the elderly experience in managing their medication therapy and their perception of medication adherence. **Methods:** Based on Husserl’s perspective, a qualitative descriptive study was conducted utilizing the phenomenological approach, specifically Interpretative Phenomenological Analysis (IPA). The data analysis followed Giorgi’s phenomenological approach and the inductive content analysis method. Approval for the study was obtained from the relevant Ethics Committee. **Results:** Themes emerged when participants described experiences about their own adherence to therapy. The following general theme emerged from the interviews and the phenomenological analysis: Generating awareness: taking therapy saves your life. It was followed by three other themes: (1) Drug therapy awareness; (2) Drug therapy in daily life; and (3) Drug therapy as a life partner. **Conclusions:** Elderly patients undergoing polypharmacological treatment emphasize the key factors for improving medication adherence, highlighting the influence of individual, motivational, and relational aspects. They express a strong desire for information and value the support of family doctors and nurses in managing therapy. Patient interviews indicate general support among elderly patients for using mobile health in pharmacological treatment, recognizing its potential and limitations.

## 1. Introduction

According to the World Health Organization’s data, by 2030, one in six people worldwide will be aged 60 or over. By 2050, forecasts suggest that the population in this age group will double, reaching 2.1 billion. Additionally, it is anticipated that the number of individuals aged 80 or older will triple between 2020 and 2050, reaching a total of 426 million [1].

While many countries classify individuals as elderly at the age of 65, this perspective is often challenged due to increasing life expectancy, improved quality of life, and higher levels of functioning among older populations [2]. The literature recommends using the age at which individuals become eligible for state pensions, yet definitions of an elderly population are multifaceted, often incorporating factors such as chronological age, shifts in social roles, and changes in capabilities [3]. The complexity of defining old age can make it difficult for clinicians practicing evidence-based medicine to assess treatment benefits for this demographic.

ISTAT data for 2022 indicate an exponential growth of the over-65s in Italy: in 2050, the share of elderly individuals will represent 34.9% of the general population, up from 23.5% in 2021 [4]. Chronic diseases represent the leading cause of death in the world, and in Italy, they are constantly increasing: 40% of the Italian population is affected by at least one chronic disease [5]. Italian data confirm this progressive growth, especially in the over-65 population. According to the 2020 report of the National Observatory on the Use of Medicines (OsMed) of the Italian Drug Agency, 65.8% of patients aged 65 years and older received prescriptions for at least five different substances during the year 2020, and about one in four (26.1%) took at least 10 different active ingredients [6,7]. Analysis of the literature suggests that the presence of comorbidities leads to an increase in the number of medications to be taken and, consequently, to medication non-adherence [8,9].

In Italy, the healthcare system operates under a universal coverage model, primarily funded through taxation. It provides access to a wide range of medical services, including prescription medications, often at low or no cost to patients, particularly for those with chronic conditions [3]. Medication adherence is supported through patient education, prescription monitoring, and a network of healthcare providers, including general practitioners and pharmacists. However, challenges remain, particularly in managing adherence in older adults with multimorbidity, due to factors such as polypharmacy, fragmented care, and the limited availability of personalized support programs [6]. 

Medication adherence is defined by the World Health Organization (WHO) as the degree to which a person’s behavior in taking medications, following a diet, and/or making lifestyle changes corresponds to recommendations agreed upon by a healthcare provider [10,11]. In the literature, increasing attention to treatment adherence, a complex phenomenon that is crucial to the success of the treatment pathway, has been observed for several years [12]. Increased medication adherence means a lower risk of hospitalization, fewer disease-associated complications, and improved safety and efficacy of treatments with reduced hospitalization costs [13,14]. In this regard, multimorbidity is a scenario that tends to become more prevalent with advancing age and requires personalized and multi-objective treatments [13]. Less than 10% of the elderly population are not prescribed medications, while 50% need 5 or more medications and 10% need 10 or more medications [15,16]. When it comes to medications, proper adherence is a key prerequisite to ensure safety and efficacy [17]. Considering the negative consequences in terms of economic burden and worsening quality of life [18,19], non-adherence to medication prescriptions is socially relevant. Medication adherence rates among the elderly vary by treatment type and geographic location but are often far from optimal [20,21]. The literature suggests that patients with various disease symptoms share key aspects of the experience of medication use, based on the physical effects of the drugs on their bodies and the social effects of medication use on the people and healthcare providers around them [22].

Given the recognized complexity of patient behavior when it comes to treatment compliance, different qualitative conceptualizations of the adherence process have been proposed to address all its facets effectively [23,24]. The literature highlights the ABC Taxonomy, which defines medication adherence as a three-stage process: initiation (taking the first dose), implementation (following the prescribed dosage), and discontinuation (stopping the medication) [12,24]. Another model, the Information–Motivation–Strategy (IMS) model, explains adherence through three key factors: information (knowledge from the patient–prescriber relationship), motivation (cognitive, social, and cultural influences), and strategy (practical plans for managing the disease) [25]. A multifaceted and personalized approach is essential for the elderly, emphasizing both social support and the patient’s motivation to begin and maintain treatment.

Health systems acknowledge the citizen/user as a crucial resource for enhancing the effectiveness and sustainability of care processes [26]. Therefore, a broader, multidisciplinary, and multidimensional vision is necessary to redefine the approach to this issue.

This approach includes both healthcare workers, with their diverse roles and expertise, and the patient as an active participant in the care process. Medication adherence has been widely studied, revealing numerous risk factors that act as barriers or facilitators [27,28,29,30,31,32,33,34,35]. However, most research focuses on adherence to specific conditions or treatments [27,28,29,30,31,32,33], with few studies addressing multimorbidity from the patient’s perspective through a phenomenological approach [34,35]. 

This study aims to explore the experiences and perceptions of medication adherence in patients over 65 with chronic conditions, focusing on polypharmacy management.

## 2. Materials and Methods

### 2.1. Study Design

This descriptive qualitative study was conducted using open-ended interviews focused on exploring the patient’s perspective and, at the same time, enabling the nurse to gain a deeper understanding of the subjects’ views in the study [36,37,38]. Husserl’s philosophical perspective was adopted using a qualitative phenomenological approach [37].

Phenomenological research is a research approach that investigates the everyday experiences of human beings, while still considering the researchers’ preconceived assumptions about the phenomenon. IPA (Interpretative Phenomenological Analysis) is an experiential methodology that best focuses on how people make sense of what happens to them, as suggested in the literature [39,40].

The EMERGE (Medication Adherence Reporting Guideline) taxonomy, designed to standardize the definition of medication adherence, was used to construct the interview questions [41]. The COREQ (Consolidated Criteria for Reporting Qualitative Research) guidelines were used to improve the study’s consistency, quality, and rigor [42] (See Supplementary Materials).

### 2.2. Study Sampling and Recruitment

Elderly patients meeting the eligibility criteria were recruited from a Primary Care Center in Rome, Italy. Elderly patients belonged to the ASL referral of the Primary Care Center chosen by the researchers. They were briefed on the study’s objectives and requested to provide informed consent for participation in the interviews. With the patients’ consent, all open-ended, one-to-one interviews were audio-recorded. The project’s principal investigator, a nurse, conducted interviews until data saturation was achieved, indicating that no new concepts or information emerged from the interviews [43]. The research nurse did not work at the Primary Care Center. 

Adhering to Sandelowski’s sampling strategy to ensure equality in data collection, the inclusion criteria were as follows [44]: patients aged 65 and above with chronic conditions undergoing polypharmacy and possessing a proficient understanding of the Italian language. Exclusions comprised patients with cognitive deficits, those without chronic conditions or not taking medications, and individuals under the age of 65.

### 2.3. Setting and Data Collection

Data were collected in a Primary Care Centre from November 2023 to January 2024. A nurse and patients in designated locations within the treatment center conducted open-ended, one-to-one interviews (Table 1). The nurse involved in the interviews was a PhD student. Patients were encouraged to describe their experiences freely and without fear of being judged. Interviews were conducted in the Primary Care Center room, and there was no barrier between the interviewee and the researcher. The gathered interviews were transcribed verbatim, and tables and grids were utilized to evaluate data saturation. Each interview lasted 40 to 60 min. The importance of confidentiality of sensitive data was explained to them. Before the interviews, socio-demographic information of the patients involved in the study was collected. The nurse introduced the topic overall, and then the questions were enunciated to the patients. 

### 2.4. Data Analysis

The data analysis adhered to Giorgi’s phenomenological approach [45] and applied the inductive process of the content analysis method [46]. Specifically, it encompassed the five phases within Giorgi’s descriptive phenomenological approach [45]: 1—gathering data (interviews); 2—reviewing the data; 3—breaking down data into segments; 4—organizing data from a disciplinary standpoint; and 5—synthesizing data to articulate the structure of the phenomenon. Each interview, facilitated by a nurse (GL, RN, PhD student), was subsequently transcribed verbatim by a second research nurse (BG, RN, MSN student) to maintain accuracy and neutrality. The transcription meticulously captured pauses, silent gestures, and the patient’s sentiments on the topic. In the second phase, the transcript underwent a reexamination to uncover the overall meaning and the individual’s perspective without biases, steering clear of data aggregation.

In the third phase, nurses (a GL, RN, PhD student and a BG, RN, MSN student) reevaluated the text to pinpoint primary “units of meaning”, representing concepts with autonomous meanings [45]. These units of meaning were then rearranged from a disciplinary standpoint and aligned with the clinical perspective. Ultimately, in the abstraction phase, similar units of meaning were grouped into macro-categories to elucidate the phenomenon’s essence, elucidated through topic-related themes and sub-themes. Two nurses (a GL, RN, PhD student and a BG, RN, MSN student) conducted the data analysis under the guidance of a senior nurse (NP, RN, PhD) with expertise in qualitative research. The researchers refrained from using qualitative analysis software. Collected interview data were structured, summarized, and analyzed using grids and tables, with continuous discussions among researchers to stay in line with the study’s goals of capturing the essence of patient experience and feelings regarding medication adherence. Data collection and analysis proceeded concurrently until reaching data saturation, where no new concepts surfaced.

To ensure the results’ credibility, dependability, confirmability, and transferability, the study adhered to the Lincoln and Guba criteria [47]. Strategies included persistent observation and data collection until saturation, triangulation of sources, methods, and researchers, constant interaction within the research team, analysis of contrary cases, discussions with colleagues, peer debriefings, mutual controls, and audit trials involving external researchers not affiliated with the study (EDS, RN, PhD, and ADL, RN, PhD students). Diaries were also maintained throughout the research process.

### 2.5. Ethics and Dissemination

The research protocol obtained approval from the Ethics Committee Lazio 1 (Ref. N 0825/2023) and adhered to the 1964 Helsinki Declaration and its subsequent amendments [48]. Informed consent was obtained from every individual participant enrolled in the study. 

Before conducting interviews, participants received information about the study’s objectives and purposes. Additionally, before each interview, nurses established a positive, trust-based relationship with participants and assessed their comprehension of the topic. All research nurses engaged in conducting interviews and analyzing data possessed significant experience in qualitative research. The data were presented in an aggregated and pseudo-anonymized format, ensuring that direct attribution to the study’s participants is not possible.

### 2.6. Patient and Public Involvement

Patients actively participated from the beginning of the data collection phase of this study. Before the interviews, patients were informed about the research questions, study design, objectives, and data analysis. The core of this qualitative study revolved around the patients’ narratives, and seeked to understand their experiences and feelings regarding their course of polypharmacy. The study’s results were shared with the patients during a patient conference held at the Primary Care Centre where the research took place. After each interview, the nurse conducted a debriefing session with the interviewee to evaluate the impact of the intervention on the patients.

## 3. Results

### 3.1. Patients Socio-Demographic and Clinical Characteristics

The socio-demographic and clinical characteristics of the patients involved in the study are described in Table 2. A total of 20 patients were included in the qualitative study. The average age of the participants was 73.75 (range 68–82), with 50% being female and the remaining 50% male. Thirty percent of the patients had primary education, 55% attended middle school, and 1% of the participants had a degree. Seventy percent of patients over 65 were married and living with a partner, while 50% lived with a child.

The most common chronic conditions in the sample were cardiovascular diseases, affecting approximately 80% of the patients. A total of 75% of the patients involved in the study took more than five medications per day. In collecting data about the number of drugs taken daily by the patient, no distinction was made regarding the type of drug taken by the patient, but only the pill number.

### 3.2. Phenomenological Analysis: Themes and Sub-Themes

Iterative coding procedures were utilized to reveal noteworthy themes within the dataset. The phenomenological analysis identified one general theme, three principal themes, and six sub-themes (Table 3, Table 4 and Table 5). These themes were derived from the analysis of the verbal transcripts of interviews. Each patient’s information, including an assigned code (P1–P20), gender (F for female, M for male), and age, were reported in brackets following each sentence, such as (P20, M, 86 yrs).

Overall, the following general theme emerged from the interviews and the phenomenological analysis was as follows: Generating awareness: taking therapy saves your life; it was followed by three themes: (1) Drug therapy awareness, (2) Drug therapy in daily life, and (3) Drug therapy as a life partner. 

## 4. Discussion

The present phenomenological qualitative study aims to investigate patients’ experiences regarding medication adherence. Understanding patients’ experiences with medication is crucial, as non-adherence to long-term medication can result in increased morbidity and mortality [49,50]. One of the main challenges for our national healthcare system and the world is reducing the prevalence of deaths caused by chronic diseases.

As suggested in the literature, the use of medications is understood through various classifications, such as intentional and unintentional adherence, or by considering the treatment phases: initiation, implementation, and discontinuation [51,52]. 

To address the issue of medication non-adherence, it is necessary to explore the perspectives and habits of patients before subjecting them to pharmacological treatment [53]. This qualitative research suggests that the adherence to medication experience in patients over the age of 65 is often influenced by psychological and social factors, such as the need to be informed about the therapy to be taken; the sometimes lack of involvement by professionals; the difficulty of taking the therapy when away from home; awareness of the risk of not taking the therapy; the patient–prescriber relationship; trust in science; and the complexity of the treatment, as indicated in the literature [54,55,56,57]. 

The interviewed patients expressed a strong desire to be well informed about their treatment, including understanding the risks and side effects of each medication. They expressed enthusiasm for their family doctor, as they felt involved in their care for the first time, fostering a sense of partnership with their healthcare provider.

Elderly patients express considerable trust in healthcare workers, emphasizing the importance of always being involved in the choice of their drug therapy. The evidence suggests that the therapeutic relationship with healthcare professionals makes the patients’ experience with medication use more meaningful [58]. The consistency in the information healthcare professionals provide to patients can play a vital role in medication adherence. Furthermore, the literature suggests that patients who report non-adherence to medication experience negative social consequences rather than negative health outcomes, and that these experiences have been normalized by healthcare providers [23].

From the conducted qualitative study, it emerges that today’s patients are increasingly well informed and understand the appropriateness of the prescribed pharmacological therapy. This reflects a shift in patient behavior, where individuals actively seek to understand the rationale behind their treatments, ensuring that medications align with their specific needs. They are also aware of the risks associated with non-adherence to therapy, such as worsening health outcomes or increased complications, and recognize the potential benefits of adhering to their prescribed regimen, which can lead to better management of their conditions and an overall improved quality of life. This growing patient awareness underscores the importance of clear communication and shared decision-making between healthcare providers and patients. As suggested in the literature, patients with chronic conditions are aware that medications contribute to their survival [59]. Despite the participants reporting good adherence, missed doses were attributed to distraction, being away from home, and timing issues, as suggested by the results of a previous qualitative study [57]. It is crucial that elderly patients understand the specific purpose of taking medications, such as prevention, symptom relief, and treatment. The literature suggests that patients often report using a medication because it has become part of their daily routine, rather than due to the necessity for a specific perceived symptom [23]. The more satisfied they are with their pharmacological treatment, the less it impacts their daily life, leading to better adherence [60]. From the interviews, it emerges that patients satisfied with their pharmacological therapy have not changed their lifestyle habits, even after starting the medication treatment.

The interviews reveal that patients actively adapt their medication regimens to align with their unique lifestyles, emphasizing the critical need for a more personalized approach in supporting their adherence and overall health management. This customization underscores the importance of understanding each patient’s daily routines, preferences, and challenges when designing treatment plans. Additionally, patients express a clear desire for future innovations in healthcare, such as the development of a single pill capable of treating multiple conditions which would simplify their medication routines, reduce the burden of polypharmacy, and enhance their quality of life. This forward-looking vision highlights the demand for medical advancements that prioritize convenience, efficiency, and patient-centered care. Another emerging element is the use of technology to support pharmacological therapy adherence. Providing reminders and alerts and facilitating communication between patients and healthcare professionals seem to be highly desired solutions for elderly patients [61]. They already declare using technology in their daily lives, such as mobile phones, Alexa, and instant messaging systems. The goal of these systems is to enhance patient outcomes by reducing the risk of medication errors and complications, supporting patients in more effectively managing their chronic conditions [61,62,63,64].

The findings of this study underscore the importance of actively listening to patients and respecting their preferences regarding drug therapy management to minimize obstacles to treatment adherence. Specifically, patients express a desire to harness technology more effectively to aid in managing their drug therapy. This aligns with the new LEAs in Italy (essential levels of care), which advocate for the nationwide implementation of information and communication tools to streamline patient care processes.

### 4.1. Implications for Future Research and Clinical Practice

Patient-reported experiences show that habits, behaviors, and patient perceptions have emerged as strong predictors of non-adherence. Studying the behavior of elderly patients from a multidisciplinary perspective can anticipate their pharmacological non-adherence. Identifying patient preferences and involving them in the process of selecting pharmacological therapy can help improve medication adherence. Future research should address specific issues related to multimorbidity and polypharmacy, as well as potential differences in beliefs and treatment management based on cultural factors and lifestyle habits. Further research could investigate the experience of adherence across cultural contexts in various disease states.

### 4.2. Limitations

The authors acknowledge the limitations of the current study. Firstly, there is a potential for the interviewed patients to have altered their responses either out of fear of judgment for non-adherence to the therapy or to align with interviewer expectations (social desirability bias). Exploring the patients’ urban or rural backgrounds could have added valuable insights to the study. The qualitative approach employed may limit the generalizability of the results to a broader population or to different populations with other cultures. Nevertheless, the utilization of Lincoln and Guba’s criteria aimed to enhance the reliability of the study results [47]. 

Conducting research in a single healthcare center may limit the generalizability of the findings, as the experiences and healthcare practices observed could be specific to that particular setting. Differences in regional healthcare systems, patient populations, and local practices may not be fully captured, potentially impacting the broader applicability of the results. Future studies could address this by including multiple centers to ensure a more diverse and representative sample, enhancing the external validity of the findings.

## 5. Conclusions

This study’s findings underscore key factors influencing medication adherence among elderly patients with chronic conditions, including the need for healthcare professionals, particularly nurses, to understand the impact of individual, motivational, and relational factors. Patients emphasize the importance of clear communication and the pivotal role of family doctors and nurses in managing therapy, particularly during hospitalization. However, adherence becomes more variable in primary care settings where patients manage medications independently, highlighting the need for improved continuity of care and support during the transition from hospital to home [65].

Themes arising from patient interviews suggest that elderly patients with chronic conditions are generally supportive of using mobile health as a tool for pharmacological therapy, recognizing its potential [66,67,68].

Future research should focus on the complexities of multimorbidity, polypharmacy, and the influence of cultural and personal perceptions on treatment adherence. The EMERGE guidelines, based on the ABC taxonomy [37], are recommended for structuring future studies, with an emphasis on the initiation phase of medication adherence. A multidisciplinary, patient-centered approach is crucial for improving adherence in this population [69].

## Figures and Tables

**Table 1:** Interviews.

INITIATION
Do you find the information you receive about your medications understandable?
What is your opinion on medications?
What concerns do you have when starting a new therapy?
What kind of support have you received to adhere to your pharmacological treatment?
IMPLEMENTATION
What is your experience with pharmacological treatment?
Do you believe you have good adherence to medications? Why?
Do you think your pharmacological treatment suits your needs, preferences, and lifestyle?
How do you manage the timing and schedules of your medications?
How would you describe the medications you take? Do you consider them effective, safe, easy to take, easy to identify, etc.?
What strategies do you use to correctly take your therapy?
DISCONTINUATION/PERSISTENCE
Are you aware of the risk involved in not taking a therapy?
Have you had a negative experience while taking medication? If yes, has this situation led you to discontinue the treatment?
What do you think would truly help you improve your adherence to treatment?

**Table 2:** Socio-demographic and clinical characteristics.

Patients’ Characteristics	n 20
Age (mean)	73.75 (68–82)
Gender	
Male	10 (50%)
Female	10 (50%)
Education	
Primary school	6 (30%)
Middle school	11 (55%)
High school	2 (10%)
Degree	1 (5%)
Marital status	
Single	2 (10%)
Married	15 (75%)
Divorced/Separated	1 (5%)
Widow	2 (10%)
Employment	
Retired	20 (100%)
Cohabitation with partner	
Yes	15 (75%)
No	5 (25%)
Cohabitation with son/daughter	
Yes	10 (50%)
No	10 (50%)
Chronic diseases	
Diabetes	5 (25%)
Stroke	4 (20%)
Hypertension	16 (80%)
Heart disease	14 (70%)
Cancer	3 (15%)
Neurodegenerative diseases	3 (15%)
Thyroiditis	6 (30%)
N. of drugs every day	
<5	6 (30%)
>5	14 (70%)

**Table 3:** Themes and sub-themes.

General Theme	Themes	Sub-Themes
Generating awareness: taking therapy saves your life.	Drug therapy awareness	Drugs help you feel better
		Technology to support medication adherence Patient involvement and knowledge
	Drug therapy in daily life	From conscious hesitancy to unconscious forgetfulness
		Innovation: between feasibility and dream
	Drug therapy as a life partner	Compulsory coexistence with illness
		Trust in science and health professionals

**Table 4:** General theme and quotations.

General Theme	Quote	Participant	Age	Gender
Generating awareness: taking therapy saves your life	“Since I started taking these medications, I definitely feel better”.	P2	74 yrs	Male
Adherence to therapy is a complex process for the patient; the main theme from the interviews is that the patient is always aware of the risks involved in not adhering to treatment. Patients are aware of the vital importance of drug therapy and the benefits that drugs bring to their health:	“Some medications I have to take do affect me, but if I forget them at home and can’t return at that exact time, then I miss that treatment, and it bothers me psychologically because I always have that thought lingering…”	P2	74 yrs	Male
	“My therapy is the only way to save myself and live a bit longer…”	P5	82 yrs	Male
	“Medications are essential for saving lives, treating various conditions, and therefore, welcome are the medications; they are well accepted…”	P3	75 yrs	Male
	“My therapy has saved my life, so it’s crucial that I take it correctly”.	P10	69 yrs	Female
	“My therapy has saved my life, so it’s crucial that I take it correctly”.	P10	69 yrs	Female
	When the patient skips the treatment, they immediately recognise the risks involved: “Then I got concerned because my triglycerides were very high, so I immediately resumed the treatment”.	P6	76 yrs	Female

**Table 5:** Themes and sub-themes and representative quotations of elderly people’s experiences.

Themes	Representative Quotations
Theme 1: Drug therapy awareness	The patient desires to receive comprehensive information regarding their drug treatment. Frequently, they find themselves insufficiently engaged in the decision-making process concerning their medication, recognizing that possessing knowledge is crucial for understanding the advantages of adhering to the prescribed treatment: *“I believe that the medications make me feel well when I take them…”* (P16, M, 68 yrs). *“Medications are crucial when recommended by one’s doctor”* (P2, M, 74 yrs). *“The medications are essential because without them, I wouldn’t be well, and I wouldn’t have survived until now, so I consider myself very fortunate that they exist…”* (P17, M, 75 yrs). *“Today with the current doctor Flavio, I have great confidence in him and so I am calm, I know serene when he recommends some drug, in fact I always tell him “I want to live…””* (P1, M, 82 yrs). *“My opinion is that they are definitely crucial to take…”* (P2, M, 74 yrs). *“Every time I ask for information, my doctor is always available to provide it”.* (P3, M, 75 yrs). Utilizing digital systems for medication adherence proves to be an effective approach in enhancing adherence and assisting patients in more effectively overseeing their health: *“When I’m at home, I use Alexa, which helps me remember the exact moment when I need to take the pill…”* (P2, M, 74 yrs). *“I use technology a lot; for instance, I use Alexa to help me remember all the pills throughout the day…”* (P13, M, 68 yrs). *“I believe that the use of technology could be helpful. I see my granddaughters always using their phones, so let’s make it useful in this regard too…”* (P17, M, 75 yrs). *“I jot down the list of medications on the notes section of my phone... I use it a lot for Facebook, WhatsApp, and video calls, so I never have any issues”.* (P18, M, 68 yrs). Identifying gaps in knowledge as a factor contributing to non-adherence continues to pose a significant challenge for multidisciplinary teams. The patient would be involved in the choice of their pharmacological therapy: *“I am a well-informed person who always tries to know everything before starting a new therapy…”* (P18, M, 68 yrs). *“Information about medications is always somewhat relative, but accurate because it comes from a competent person. I also gather information on my own”.* (P20, F, 74 yrs). *“My doctor always informs me about the therapy I need to take”.* (P2, M, 74 yrs). *“At times, I’ve been prescribed medications without being told precisely what they were for, but then I researched and understood why I had to take them”.* (P3, M, 75 yrs).
Theme 2: Drug therapy in daily life	Taking medication correctly is an instrumental activity of daily living (IADL) [49]. The patient has integrated pharmacological therapy into their daily life and has not changed their previous habits: *“Since I started these therapies, I have never changed my lifestyle habits. I continue to take care of my plants and manage the vegetable garden and the garden”.* (P6, F, 77 yrs). *“These therapies have not influenced my habits much, also because I try to manage them in a way that respects my daily activities”.* (P7, F, 75 yrs). *“The pills do not pose problems for me when I need to start an activity because, generally, I organize myself. I spend my free time gardening and reading poems”.* (P15, M, 79 yrs). Sometimes patients show conscious hesitation; sometimes, they simply forget to take the therapy. Patients frequently encounter challenges in adhering to their treatment regimen, such as being frequently away from home, inadvertently falling asleep before administering their medication, struggling to recall whether they have already taken it, experiencing anxiety surrounding the therapy, or becoming confused about the dosage schedule. *“Sometimes it has happened to me that I might forget it because I am away from home, especially when I have a very tight schedule for the treatment”.* (P2, M, 74 yrs). *“I often find that I move my medication times around so that I can take them at home, maybe I’m busy with other activities and I forget to bring them or take them at all”.* (P14, F, 75 yrs). *“When I leave home and spend many hours outside, I might forget to take the medication blister with me, and it slips my mind. For instance, to prevent this, lately, I’ve been carrying a small box with spare tablets”.* (P7, F, 75 yrs). *“My doctor, Flavio, told me that I should not take a double dose if, for instance, I forget to take it because it could be dangerous”.* (P9, M, 70 yrs). Patients often seek innovation in strategies to enhance their therapy adherence, and they frequently dream of something that is feasible in their everyday reality. Patients often wish to share the burden of their illness with other patients who suffer in the same way; moreover, they would like to have therapies tailored to their physical condition, such as the monopill. *“It would be nice to have meetings with other patients who suffer from the same condition to share experiences and feel less alone, perhaps understanding the difficulties in adhering to one’s therapy”.* (P20, F, 74 yrs). *“Therapies should be personalized and not the same for everyone because our bodies can react differently than someone else’s”.* (P19, F, 72 yrs). *“The solution could be the single pill, as I heard on TV some time ago… it should treat you for multiple conditions, and it’s a single drug to take”.* (P16, M, 68 yrs). *“I wish there was a single drug that could cure the disease directly…”* (P7, F, 75 yrs). *“I think it would be helpful to have the monopill because since I’ve been taking the drug combinations, I’ve been doing very well…”* (P4, M, 81 yrs).
Theme 3: Drug therapy as a life partner	Pharmacological therapy is integrated into the lives of these patients: *“I usually coordinate taking the therapy with mealtime, so breakfast, lunch, and dinner”.* (P19, F, 72 yrs). *“I always check the clock in my kitchen and realize it’s the right time to take the medications”.* (P20, F, 74 yrs). *“My wife and I help each other take the tablets every day because we both have to take a lot of them…”* (P8, M, 76 yrs). Patients constantly live with the compulsive thought of the weight of the disease afflicting them: *“I have Parkinson’s, which is now compromising my life; I can’t engage in physical activities like before… when I received the diagnosis, my world collapsed”.* (P2, M, 74 yrs). *“Not having the disease would be the best thing*… *everything else is just something that can support me… the illness is always a burden for me”.* (P3, M, 75 yrs). *“The emotions I felt in the hospital when I received the cancer diagnosis were very negative”.* (P12, M, 74 yrs). Patients demonstrate significant trust in science and healthcare professionals: *“I really trust my doctor, I don’t worry, I trust”* (P11, F, 70 yrs). *“When you have a disease, you try in every way to seek help and completely entrust yourself to science, so I trust specialists and my doctor”.* (P6, M, 77 yrs). *“My doctor always informs me about the therapy I need to take”.* (P2, M, 74 yrs). *“I trust the professional, the doctor…I have a lot of faith in science”.* (P2, M, 74 yrs). *“The support was very present in the hospital because I had nurses who helped me with everything…”* (P6, F, 77 yrs).

## Data Availability

Data are contained within the article.

## Supplementary Materials

The following supporting information can be downloaded at: https://www.mdpi.com/article/10.3390/healthcare12191925/s1, Supplementary File S1: Consolidated criteria for reporting qualitative studies (COREQ): 32-item checklist.
