# Supplementary material for: A Phenomenological Approach to Medication Adherence in Elderly Patients: A Qualitative Study
Source: Healthcare (Basel). 2024 Sep 25;12(19):1925. doi: 10.3390/healthcare12191925 (PMC11476112; doi:10.3390/healthcare12191925)
Supplement: Supplementary file 1 [file healthcare-12-01925-s001.zip › healthcare-3186531-supplementary.pdf]

Supplementary File S1

Consolidated criteria for reporting qualitative studies (COREQ): 32-item checklist for manuscript in title:

"A phenomenological approach to medication adherence in elderly patients: a qualitative study"

No	Item	Guide questions/description	Response
**Domain 1: Research team and reflexivity**			
Personal Characteristics			
1.	Interviewer/facilitator	Which author/s conducted the interview or focus group?	Done<br>It is mentioned in the method.
2.	Credentials	What were the researcher's credentials? *E.g. PhD, MD*	Done<br>It is mentioned in the authors information in title page.
3.	Occupation	What was their occupation at the time of the study?	Done<br>It is mentioned in the authors information in title page.
4.	Gender	Was the researcher male or female?	We added in the authors information in title page.
5.	Experience and training	What experience or training did the researcher have?	It can be obvious from authors information.
Relationship with participants			
6.	Relationship established	Was a relationship established prior to study commencement?	No
7.	Participant knowledge of the interviewer	What did the participants know about the researcher? e.g. *personal goals, reasons for doing the research*	At the beginning of each interview, we explained the individual goals and reasons for doing the research. It is mentioned in the method.
8.	Interviewer characteristics	What characteristics were reported about the interviewer/facilitator? e.g. *Bias, assumptions, reasons and interests in the research topic*	Interviewers didn't report their own assumptions for not giving direction to the interviewees. But, we explained the personal goals, reasons for doing the research.
**Domain 2: study design**			
Theoretical framework			
9.	Methodological orientation and Theory	What methodological orientation was stated to underpin the study? *e.g. grounded theory, discourse analysis, ethnography, , content analysis*	*phenomenology*<br>It is mentioned in the method section
Participant selection			
10.	Sampling	How were participants selected? *e.g. purposive, convenience, consecutive, snowball*	*Purposive*<br>It is mentioned in the method section.
11.	Method of approach	How were participants approached? e.*g. face-to-face, telephone, mail, email*	*face-to-face*<br>It is mentioned in the method section.
12.	Sample size	How many participants were in the study?	20<br>It is mentioned in the method section.

13.	Non-participation	How many people refused to participate or dropped out? Reasons?	0
Setting			
14.	Setting of data collection	Where was the data collected? e*.g. home, clinic, workplace*	*workplace*<br>It is mentioned in the method section.
15.	Presence of non-participants	Was anyone else present besides the participants and researchers?	No, only participants and researchers. It is mentioned in the method section.
16.	Description of sample	What are the important characteristics of the sample? *e.g. demographic data, date*	It was presented in Table2: "Socio-demographic and clinical characteristics".
Data collection			
17.	Interview guide	Were questions, prompts, guides provided by the authors? Was it pilot tested?	It is mentioned in the method section. Wasn't a pilot test.
18.	Repeat interviews	Were repeat interviews carried out? If yes, how many?	No<br>It is mentioned in the method section.
19.	Audio/visual recording	Did the research use audio or visual recording to collect the data?	Yes<br>It is mentioned in the method section.
20.	Field notes	Were field notes made during and/or after the interview or focus group?	Yes<br>It is mentioned in the method section.
21.	Duration	What was the duration of the interviews or focus group?	Each interview lasted 40 to 60 minutes. It is mentioned in the method section.
22.	Data saturation	Was data saturation discussed?	It is mentioned in the method section.
23.	Transcripts returned	Were transcripts returned to participants for comment and/or correction?	No. It is mentioned in the method section.
**Domain 3: analysis and findings**			
Data analysis			
24.	Number of data coders	How many data coders coded the data?	There were two data coders in this study.
25.	Description of the coding tree	Did authors provide a description of the coding tree?	All cods consisted of Issues, Themes and Sub-themes which have been provided in Table 4,5 and 6.
26.	Derivation of themes	Were themes identified in advance or derived from the data?	It is mentioned in the method section
27.	Software	What software, if applicable, was used to manage the data?	It is mentioned in the method section
28.	Participant checking	Did participants provide feedback on the findings?	No. It is mentioned in the method section.
**Reporting**			
29.	Quotations presented	Were participant quotations presented to illustrate the themes / findings? Was each quotation identified? e*.g. participant number*	Done
30.	Data and findings consistent	Was there consistency between the data presented and the findings?	Done
31.	Clarity of major themes	Were major themes clearly presented in the findings?	Done

32.	Clarity of minor themes	Is there a description of diverse cases or discussion of minor themes?	The descriptive findings is presented based on “Themes” not “Sub themes”. Because of limitation in word number of article.
